# Varying congruence among spatial patterns of vascular plants and vertebrates based on habitat groups

**DOI:** 10.1002/ece3.3348

**Published:** 2017-09-20

**Authors:** Haigen Xu, Yun Cao, Mingchang Cao, Jun Wu, Yi Wu, Zhifang Le, Peng Cui, Jiaqi Li, Fangzhou Ma, Li Liu, Feilong Hu, Mengmeng Chen, Wenjun Tong

**Affiliations:** ^1^ Nanjing Institute of Environmental Sciences Ministry of Environmental Protection of China Nanjing China; ^2^ Department of Biology Nanjing University Nanjing China

**Keywords:** biodiversity, concordance, correlation, habitat, hypothesis, spatial linear model, species richness

## Abstract

Proxies are adopted to represent biodiversity patterns due to inadequate information for all taxa. Despite the wide use of proxies, their efficacy remains unclear. Previous analyses focused on overall species richness for fewer groups, affecting the generality and depth of inference. Biological taxa often exhibit very different habitat preferences. Habitat groupings may be an appropriate approach to advancing the study of richness patterns. Diverse geographical patterns of species richness and their potential mechanisms were then examined for habitat groups. We used a database of the spatial distribution of 32,824 species of mammals, birds, reptiles, amphibians and plants from 2,376 counties across China, divided the five taxa into 30 habitat groups, calculated Spearman correlations of species richness among taxa and habitat groups, and tested five hypotheses about richness patterns using multivariate models. We identified one major group [i.e., forest‐ and shrub‐dependent (FS) groups], and some minor groups such as grassland‐dependent vertebrates and desert‐dependent vertebrates. There were mostly high or moderate correlations among FS groups, but mostly low or moderate correlations among other habitat groups. The prominent variables differed among habitat groups of the same taxon, such as birds and reptiles. The sets of predictors were also different within the same habitat, such as forests, grasslands, and deserts. Average correlations among the same habitat groups of vertebrates and among habitat groups of a single taxon were low or moderate, except correlations among FS groups. The sets of prominent variables of species richness differed strongly among habitat groups, although elevation range was the most important variable for most FS groups. The ecological and evolutionary processes that underpin richness patterns might be disparate among different habitat groups. Appropriate groupings based on habitats could reveal important patterns of richness gradients and valuable biodiversity components.

## 1 INTRODUCTION

Understanding spatial patterns in species richness is central to ecology and biodiversity conservation (Gaston, 2000; Kreft & Jetz, 2007; Rohde, 1992). However, information about species richness is often lacking for the majority of biological taxa on the earth (Costello, May, & Stork, 2013; Westgate, Barton, Lane, & Lindenmayer, 2014). Currently, conservation strategies often assume that congruent patterns of diversity occur among different taxonomic groups (Lamoreux et al., 2006), and further adopt indicator groups as surrogates to represent multitaxa diversity patterns (Mac Nally et al., 2002; van Weerd & Haes, 2010; Westgate et al., 2014). Identification and application of indicator groups can greatly facilitate biodiversity monitoring and conservation planning (Duan et al., 2016). Some studies indicate that spatial patterns of species richness often coincide among different taxa (Howard et al., 1998; Lamoreux et al., 2006; Qian & Kissling, 2010; Qian & Ricklefs, 2008). However, little congruence occurs among different taxa in other studies (Grenyer et al., 2006; van Jaarsveld et al., 1998; Orme et al., 2005; Prendergast, Quinn, Lawton, Eversham, & Gibbons, 1993). Such differences may result from low taxonomic coverage, different spatial scales (Grenyer et al., 2006; Qian & Kissling, 2010), and varying species traits. Thus, the efficacy of surrogates deserves further verification despite its wide use in biodiversity conservation.

Spatial patterns of species richness are the intriguing phenomena created by biotic and abiotic factors (Rahbek & Graves, 2001). Many hypotheses have been proposed to explain patterns of species richness, for example, the energy hypothesis, the environmental stability hypothesis, and the habitat heterogeneity hypothesis (Xu et al., 2015). The energy hypothesis insists that water‐energy dynamics, ambient energy, and productivity are responsible for geographical‐richness patterns (Francis & Currie, 2003; Hawkins et al., 2003; Luo et al., 2012). The environmental stability hypothesis posits that a stable environment could be favorable to increase in species richness (Luo et al., 2012; Qian & Ricklefs, 2004). The habitat heterogeneity hypothesis asserts that diverse habitats lead to higher species richness (Kreft & Jetz, 2007). However, the explanatory power of different hypotheses and their relative roles in explaining variation of species richness among different groups require more and robust tests (Kreft & Jetz, 2007; Rahbek & Graves, 2001).

China is one of several “mega‐diversity” countries in the world (Tang, Wang, Zheng, & Fang, 2006; Xu et al., 2008). It covers a variety of ecosystems, such as forests, grasslands, deserts, wetlands, and farmlands, and exhibits extremely high species richness. Currently, the unified mechanism associated with diverse environmental determinants cannot often be proposed when explaining species richness (Carnicer & Diza‐Delgado, 2008). Analyses have previously focused on overall species richness for fewer groups, which affects the generality and depth of inference (Xu et al., 2015). Biological taxa often exhibit very different habitat preferences, which further exert the effect on spatial patterns of specific taxa (Gelderblom, Bronner, Lombard, & Taylor, 1995). Habitat groupings may be an appropriate approach to advancing the study of the species‐richness patterns (Xu et al., 2015). For the last decades, considerable progresses have been made in studying spatial‐richness patterns of a single or several taxa in China (Lin et al., 2009; Qian, 2013; Qian & Kissling, 2010; Wang, Fang, Tang, & Lin, 2011). However, quantitative analysis based on habitat groups of all vascular plants and vertebrates across China is rare except our previous study of mammal and bird species richness (Xu et al., 2015).

In this study, we used a comprehensive database of the geographical distribution of 32,824 species of wild vascular plants, amphibians, reptiles, resident birds, and mammals from 2,376 counties in the terrestrial and inland water ecosystems of China, and partitioned the five taxa into 30 habitat groups based on plant growth forms or animal habitats, to elucidate the diverse geographical patterns in species richness of 30 habitat groups and their potential mechanisms. Specifically, we examined geographical variation in, and congruence of, species richness among taxa and habitat groups, respectively, tested energy, environmental stability and habitat heterogeneity hypotheses explaining patterns of species richness, and assessed the implications of our findings for biodiversity conservation at the national scale.

## 2 METHODS

### 2.1 Species‐richness data

We considered wild vascular plants, amphibians, reptiles, resident birds, and mammals in the terrestrial and inland water ecosystems of China. In spite of the important role in ecosystem services, other species such as invertebrates and microorganisms were not considered as their information is largely undocumented. Marine species and cultivated or bred species were not included. We used a comprehensive database of the geographical distribution for 590 mammal species, 849 resident bird species, 460 reptile species, 406 amphibian species, and 30,519 vascular plant species from 2,376 counties across China (Xu, Cao, Wu, & Ding, 2013; Xu et al., 2015, 2016). We adopted “county” as the basic assessment unit in this study (2,376 counties across China) (Xu et al., 2015, 2016). Data on species distribution in counties were collected from (1) species distribution information from over 1,000 literatures on fauna and flora across China; (2) record information of specimens in herbaria of the Chinese Academy of Sciences and relevant universities; and (3) field surveys in different regions (Xu et al., 2013, 2015, 2016). According to the IUCN Red List Categories and Criteria (Version 3.1), threatened species are those species that are critically endangered, endangered, or vulnerable. We divided the above biological taxa into habitat groups according to plant growth forms or animal habitats. The habitat types of plants and vertebrates were compiled based on Flora of China (Wu, Raven, & Hong, 1994–2006) and Fauna Sinicae (Editorial Committee of Fauna Sinicae, 1978–2012). Generalist species were categorized into several habitat groups if they occupy more than one habitat type (Xu et al., 2015). For animals, habitat groups might not be exclusive. For instance, some vertebrate species might occur in two or more habitat groups. Such species were categorized into several habitat groups based on the habitats they actually occur.

Species richness was the total number of species present in a county. Mean species richness across vascular plants and vertebrates was defined as (eq. (1)):

(1) $$\{\left[\left(x_{1}-{\text{Min}}_{1}\right)/\left({\text{Max}}_{1}-{\text{Min}}_{1}\right)\right]+\frac{1}{4}\sum_{i=2}^{5}\left(x_{i}-{\text{Min}}_{i}\right)/\left({\text{Max}}_{i}-{\text{Min}}_{i}\right)\}/2$$

where *x*_*i*_ is the number of *i* species in a county; Max_*i*_ and Min_*i*_ are the maximum and minimum number of *i* species in all counties, respectively; for vascular plant *i *=* *1, mammal *i *=* *2, resident bird *i *=* *3, reptile *i *=* *4, and amphibian *i *=* *5.

Mean species richness across habitat groups was defined as (eq. (2)):

(2) $$\frac{1}{N}\sum_{i=1}^{N}\left(x_{i}-{\text{Min}}_{i}\right)/\left({\text{Max}}_{i}-{\text{Min}}_{i}\right)$$

where *x*_*i*_ is the number of species of *i* habitat group in a county; Max_*i*_ and Min_*i*_ are the maximum and minimum number of species of *i* habitat group in all counties, respectively; *i *=* *1, 2,…, *N*, and *N* is the number of habitat groups.

### 2.2 Environmental variables

Species richness at the broad scale is highly correlated with environmental factors. The energy hypotheses (water‐energy dynamics, ambient energy, and productivity), the environmental stability hypothesis, and the habitat heterogeneity hypothesis were tested (Xu et al., 2015, 2016). Nineteen environmental variables were used to analyze species‐richness gradients: (1) mean annual precipitation; (2) precipitation of the wettest quarter; (3) precipitation of the driest quarter; (4) mean annual dryness; (5) mean annual temperature; (6) maximum temperature of the warmest month; (7) minimum temperature of the coldest month; (8) annual potential evapotranspiration; (9) annual actual evapotranspiration; (10) net primary productivity; (11) normalized difference vegetation index; (12) mean diurnal range; (13) temperature seasonality; (14) temperature annual range; (15) precipitation seasonality; (16) elevational range; (17) mean elevation; (18) main land cover type; and (19) number of land cover types (Xu et al., 2015, 2016). Data on these environmental variables were obtained from public sources (Xu et al., 2015, 2016).

### 2.3 Correlation analysis

Counties in China vary in size (mean: 3,908.7 km^2^; standard deviation: 9,287.6 km^2^), which might have effects on species richness. We regressed species richness on county area (both variables were log_10_‐transformed) and obtained residuals of species richness (Lamoreux et al., 2006). We examined the relations between the residuals of species richness of vascular plants, amphibians, reptiles, resident birds, and mammals with area. However, the close relation between residuals of species richness and area often occurs. Therefore, the residuals of species richness were used for further analysis to avoid the effects of area (Qian & Ricklefs, 2008). We calculated the pairwise Spearman (two‐sided) correlation coefficient (*r*) for residuals of overall species richness and species richness of habitat groups. Spatial autocorrelation may lead to inflated estimates of the degrees of freedom in significance tests (Diniz‐Filho, Bini, & Hawkins, 2003). To remove this problem, we used Dutilleul's modified *t* test (Dutilleul, 1993) to calculate the *p*‐value for the statistical significance test of correlation coefficient based on geographically effective degrees of freedom (Grenyer et al., 2006; Qian & Ricklefs, 2008). Correlation coefficient and *p*‐value were calculated using the software “Spatial Analysis in Macroecology” (SAM) (Rangel, Diniz‐Filho, & Bini, 2010) and software Mod_t_test (http://adn.biol.umontreal.ca/~numericalecology/old/mod_t_test.html). A very high correlation was defined as *r* ≥ .9; a high correlation as .7 ≤ *r* < .9; a moderate correlation as .5 ≤ *r* < .7; a low correlation as *r* < .5; and a very low correlation as *r* < .2. We also used Dutilleul's modified *t* test with control of environmental variables to remove the effects of environmental variables.

### 2.4 Multivariate models

We used multivariate models to test hypotheses explaining species‐richness patterns as follows (Xu et al., 2015).

#### 2.4.1 Variable selection

First, we conducted Spearman correlation analysis between any two variables in each hypothesis to reduce multicollinearity. Moreover, we calculated the deviance of variables in univariate regression models. If the correlation coefficient between variables was >0.7, we considered these variables strongly intercorrelated. The variables that explained more deviance in univariate regression models were then kept (Benitez‐Lopez, Vinuela, Hervas, Suarez, & Garcia, 2014; Graf, Bollmann, Suter, & Bugmann, 2005; Kreft & Jetz, 2007). Thus, we selected a set of variables from each hypothesis for further analysis. In the second step, we carried out the hierarchical partitioning analysis based on the combination of selected predictors from each hypothesis with an aim to select the predictors that exert the most independent effects on the residuals of species richness (Mac Nally, 2002). During the hierarchical partitioning analysis, we considered all possible models in a hierarchical multivariate regression setting to collectively identify most possible predictors. We calculated the increased goodness‐of‐fit in each model with a particular variable compared to the equivalent model without this particular variable, and got the average value of the improvement in the fit across all possible models with this particular predictor included (Benitez‐Lopez et al., 2014). Thus, we got a list of predictors as well as their independent and joint effects on the residuals of species richness (Chevan & Sutherland, 1991; Mac Nally, 2000). We launched a 1,000‐randomization procedure to verify the statistical significance of the independent effects of each predictor that was called a z‐score (Mac Nally, 2002). When *p* is less than .05, *z*‐score greater than or equal to 1.65 is considered statistically significant. Finally, we selected the top six predictors based on z‐score, as they had obviously larger independent effects than other variables and excluded the multicollinearity.

#### 2.4.2 Model selection

First, we adopted generalized linear models (GLM) to establish a set of candidate models that cover all possible combinations of six core predictors (Jetz & Rahbek, 2002). Based on Akaike's information criterion (AIC), we chose the best‐fit model from the candidate models (Rangel et al., 2010). The model with the lowest AIC is considered as the best‐fit model. Second, we constructed spatial linear models (SLMs) (Kreft & Jetz, 2007) for the best models identified by GLM in the first step so that inflation of type I errors and invalid parameter estimate owning to spatial autocorrelation were avoided (Jetz & Rahbek, 2002). Simultaneous autoregressive (SAR) models were employed to account for spatial autocorrelation. Spatial error models with a lag distance of 100 km generally accounted best for the spatial structure in the data set based on the minimum value of AIC (Xu et al., 2015). By testing *z* value for its significance, we verified the contribution of each predictor to the residuals of species richness in the best‐fit SLM (Jetz & Rahbek, 2002). Third, we compared multivariate regressions of six predictors with that of 19 predictors so as to examine the robustness of six‐predictor best‐fit GLM and SLM (Jetz & Rahbek, 2002).

Species richness, areas, and environmental variables were log10‐transformed in all analyses unless otherwise stated. Statistical analyses were carried out using the software packages R, version 2.15 (Mac Nally, 2002; R Development Core Team, 2012) unless otherwise stated.

## 3 RESULTS

### 3.1 Congruence among and between habitat groups

**Figure 1:**
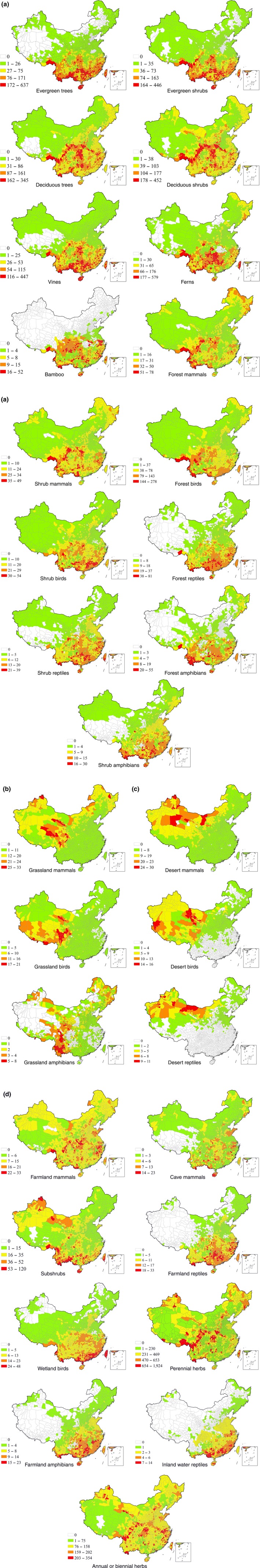
Spatial distribution in species richness of habitat groups of vascular plants, mammals, resident birds, reptiles, and amphibians in China. (a) forest‐ and shrub‐dependent groups; (b) grassland‐dependent vertebrates; (c) desert‐dependent vertebrates; and (d) other groups. Red areas are hotspots defined as the richest 5% of county areas for plant and vertebrate richness

Species richness of vascular plants, mammals, and resident birds was higher in the South than in the North and higher in the mountains than in the plains (Xu et al., 2015, 2016). Amphibians and reptiles were mainly distributed in the Qinling Mountains and further south and the eastern part of the Qinghai‐Tibetan Plateau and to the east of the plateau. Based on the combined richness data of vascular plant and vertebrate species covering different plant growth forms or animal habitats, we could assess the spatial prevalence of these taxa partitioned among 30 different habitat groups (Figure 1). We found that one major group [i.e., forest‐ and shrub‐dependent (FS) groups] had peaks of diversity around mountains located in the Qinling Mountains and further south, and the southeast section of Mount Everest—the Hengduan Mountains and further east (Figure 1a). We also found some minor groups, such as grassland‐dependent vertebrates and desert‐dependent vertebrates. Grassland‐dependent vertebrates were primarily concentrated in the Altai Mountains, the Qilian Mountains, the Hengduan Mountains, and the Minshan Mountains in western China (Figure 1b). Desert‐dependent vertebrates were mainly distributed around the Altai Mountains, the Tian Shan Mountains, the Qilian Mountains, and the Helan Mountains in northwestern China (Figure 1c).

We assessed the congruence among and between the five large taxa and different habitat groups to quantify the generality of patterns and potential processes. For overall species richness, although all pairwise Spearman's correlations were positive and significant, the cross‐taxon correlation varied markedly (Table 1). There was a high positive correlation (*r* = .82) between amphibians and reptiles; moderate correlations (.50 < *r* < .66) between plants and mammals, between plants and reptiles, between plants and amphibians, and between amphibians and mammals; and low correlations (*r* < .46) between the remaining taxa, especially between birds and all other taxa.

**Table 1:** Spearman's correlations between overall species richness of vascular plants and vertebrates. This analysis was based on data of the spatial distribution of 30,519 wild vascular plants, 406 amphibians, 460 reptiles, 849 resident bird, and 590 mammal species from 2,376 counties of China. Data of residuals of species richness were used to remove the effects of area. Spearman's correlation coefficient (Dutilleul's modified *t* test) was calculated. ***: *p* < .001, *n* = 2,376

	Vascular plants	Amphibians	Reptiles	Resident birds
Amphibians	.601***			
Reptiles	.501***	.818***		
Resident birds	.301***	.375***	.428***	
Mammals	.659***	.52***	.455***	.277***

**Figure 2:**
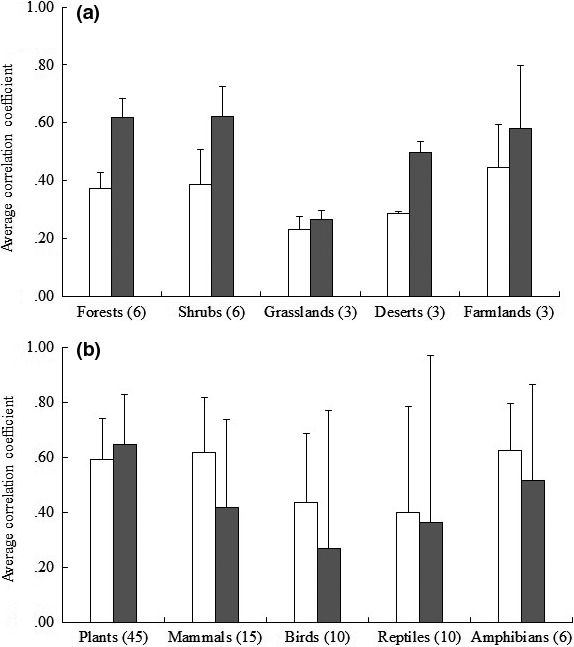
Average correlations among habitat groups (mean ± *SD*). (a) Among the same habitat groups of vertebrates; (b) among habitat groups of a single taxon. Pairwise Spearman's correlation coefficient between species richness of habitat groups was calculated using Dutilleul's modified *t* test. Residuals of species richness were used to remove the effects of area (gray bars) and the effects of area and environmental variables (open bars). Values in parentheses refer to the number of pairwise comparisons between habitat groups

We examined correlations between the species richness of habitat groups. There were mostly high or moderate (*r* > .50) correlations among FS groups, but mostly low or moderate correlations among other habitat groups (Table S1). Average correlations among the same habitat groups of vertebrates and among habitat groups of a single taxon were low or moderate (Figure 2), especially for grassland and desert habitat groups. It means that grassland‐ or desert‐dependent species have different ecological traits and exhibit different spatial patterns in particular when compared with FS groups.

### 3.2 Mechanism of spatial‐richness patterns

**Figure 3:**
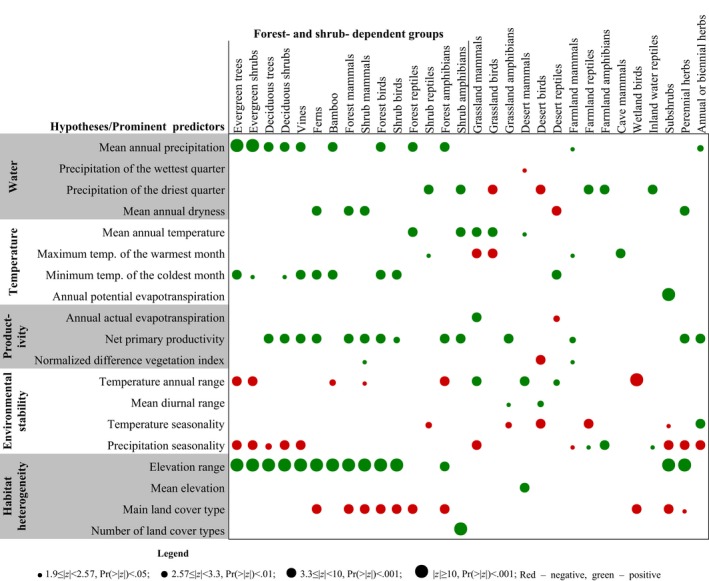
SLM multivariate models for residuals of species richness of habitat groups of vascular plants and vertebrates. Six core predictors that explained most of the variance of the residuals of species richness of each habitat group were identified by univariate regression models and hierarchical partitioning. The best multivariate model for the residuals of species richness was established using multivariable GLM regression based on AIC value. To avoid inflation of type I errors and invalid parameter estimate due to spatial autocorrelation, we performed SLM multivariate regression. All continuous variables were log10‐transformed. Red color in dots indicates negative effect and green color positive. The sets of prominent predictors (some of the six core predictors for habitat groups were not shown due to their insignificance for SLMs) of species richness differed strongly among habitat groups, although elevation range was the most important predictor for most FS groups

We used SLMs to test the five main hypotheses. We established SLM multivariate regressions for habitat groups. The six core predictors together explained 47%–89% of the variance of species richness of habitat groups (some of the six core predictors for habitat groups were not shown in Figure 3 due to their insignificance for SLMs) (Tables S2 and S3). When considering all 19 environmental variables, the change in model fit was small (Δ*r* ^2^ ranging between 0 and .06). Therefore, we are confident of the robustness of these best models. Based on these best models, we identified elevation range as the most important variable when explaining the variance in species richness of most FS groups (except reptiles and shrub amphibians), subshrubs, and perennial herbs across China (Figure 3). We also found the broad support for the ambient energy or temperature hypothesis, the energy availability hypothesis, and the environmental stability hypothesis. Energy availability and its variability were prominent variables for most habitat groups.

However, the sets of prominent variables of species richness among habitat groups were mostly different and diverse. The prominent variables differed among habitat groups of the same taxon, such as birds, reptiles, and amphibians (Figure 3). The sets of predictors were also different within the same habitat, such as forests, shrubs, grasslands, and deserts (Figure 3). It indicates that each hypothesis plays a different role in shaping species‐richness patterns of habitat groups.

## 4 DISCUSSION

This study indicated varying congruence in species richness of habitat groups and the highly complex and various interplay of environmental factors that underpin them. The correlations between the five large taxa in our study were lower than those of previous studies at the global level (Grenyer et al., 2006; Lamoreux et al., 2006; Qian & Ricklefs, 2008). It is consistent with the conclusion that cross‐taxon‐richness correlations are weaker at the local scale (Wolters, Bengtsson, & Zaitsev, 2006), as we made the analysis based on the county in China with the average area of 3,908.7 km^2^, which is much smaller than the assessment units at the global level. Besides, correlations among FS groups were high. This finding is similar to the result in Guinea where there is high congruence for richness patterns between forest birds and mammals (Burgess, Klerk, Fjeldså, Crowe, & Rahbek, 2000). However, correlations of species richness among all other habitat groups in this study were mostly low or moderate. In particular, low correlations were found between birds and all other taxa. Similar finding is also found in India: Frogs and lizards were not correlated with birds as a whole in northeast India except certain bird subgroups (Pawar, Birand, Ahmed, Sengupta, & Raman, 2007). It may result from the assumption that adaptation to the same habitat likely leads to increased average correlations between vertebrates in the same habitat (Figure 2a) and specialization in different habitats likely results in decreased average correlations between habitat groups in a single vertebrate taxon (Figure 2b), when environmental variables are considered. The average correlations among the same habitat groups of vertebrates in this study (Figure 2) were lower than those of previous studies, both at the local (1,693.4 km^2^) and provincial scale (345,516.4 km^2^) in China (Qian & Kissling, 2010). It might result from the fact that our habitat groupings differentiate between species groups with distinct ecological traits and distributions. Previous studies across a variety of taxonomic groups, natural ecosystems, and spatial scales have reported low congruence between taxa or different groups (Westgate et al., 2014).

Surrogate taxa are used widely to represent attributes of other taxa for which data are sparse or absent (Sutcliffe, Pitcher, Caley, & Possingham, 2012). Because biodiversity survey and monitoring is resource intensive, understanding and management of biodiversity rely on the availability of effective surrogates. Biodiversity surrogates provide a tractable and frequently used alternative to comprehensive monitoring or assessment of multiple taxa (Sarkar & Margules, 2002; Westgate, Tulloch, Barton, Pierson, & Lindenmayer, 2017). However, surrogacy relationships vary across spatial and temporal scales (Heino, 2014; Tulloch et al., 2016; Westgate et al., 2014) and may be weaker when examined at smaller scales compared with broader scales (Barton et al., 2014; Westgate et al., 2017). Ilg and Oertli (2017) assessed the effectiveness of amphibians as a surrogate for dragonflies, aquatic beetles, aquatic gastropods, and aquatic plants that occur in the same freshwater ecosystems in 89 ponds in Switzerland, and found that amphibians were not an effective surrogate for these four taxa. Sutcliffe et al. (2012) assessed the ability of any taxon to adequately represent others, using samples for 11 phyla distributed across 1,189 sites sampled from the seabed of Australia's Great Barrier Reef, and found that no taxonomic group was a particularly good surrogate for others. We also showed that average correlations among the same habitat groups of vertebrates and among habitat groups of a single taxon were low or moderate, except correlations among FS groups. Thus, the wide use of surrogate taxa or groups without any further verification should receive critical review (Sutcliffe et al., 2012).

Westgate et al. (2017) suggested that investigation of richness and composition simultaneously is a useful method to help practitioners identify robust biodiversity surrogates. Congruence in species composition tests the correlation between two distance matrices (Westgate et al., 2017). Through complementarity analysis, Xu et al. (2017) selected 564 optimized monitoring sites (counties) which were complementary to each other to ensure that maximum species are covered while the total number of sites is minimized. We found that overlaps between the optimized monitoring sites of any two taxa were very low, ranging between 8.7% and 20.1%. Westgate et al. (2014) found that congruence in species composition was low below 10^3^ km^2^, suggesting that at these fine spatial scales, complementarity‐based metrics applied to single taxa are unlikely to be broadly representative of biodiversity. In contrast, Bilton, Mcabendroth, Bedford, and Ramsay (2006) used data from 46 ponds in two regions of the U.K. to explore the performance of macroinvertebrate taxa as surrogates and found that all four taxa (Chironomidae, Coleoptera, Gastropoda, and Trichoptera) show >70% congruence with the pattern of community similarity between sites, that is consistent result within and between regions. They concluded that single taxonomic groups can perform consistently as indicators of community similarity between ponds (Bilton et al., 2006). Therefore, consistent methods for identifying surrogates based on complementarity between distinct taxonomic groups are urgently needed (Rodrigues & Brooks, 2007; Westgate et al., 2017).

According to the studies of Jetz and Rahbek (2002) and Kreft and Jetz (2007), we analyzed the relative importance of variables in explaining species‐richness gradients. The higher z‐score of a variable shows its more dominant effect on species‐richness gradients (Xu et al., 2016). The role of each variable and their combinations in explaining species‐richness gradients differed among habitat groups. The most important environmental determinant of species richness for FS groups was mostly elevation range. According to UNEP‐WCMC (2002), mountains account for 48% of China's total terrestrial area (Tang et al., 2006). FS groups are mainly distributed in the mountainous regions. Compared to other ecosystems, mountainous regions exhibit distinct elevation range and thus create diverse niches for species formation and specialization (Xu et al., 2015). However, elevation range was not a significant predictor for most non‐FS groups (Figure 3). Some environmental variables, such as precipitation of the driest quarter, maximum temperature of the warmest month, and temperature seasonality, become prominent in the non‐FS habitat groups and deserve more attention in the conservation decision for such habitat groups. It indicates the different roles of each hypothesis to play in explaining species‐richness gradients of habitat groups.

Spatial patterns in species richness of habitat groups were not only attributed to climate, habitat heterogeneity, productivity, or environmental stability, but also dependent on species ecological and evolutionary traits. Using Dutilleul's modified *t* test (Dutilleul, 1993), we examined the potential ecological and evolutionary mechanisms after the effects of these environmental variables were removed (Table S4). When the effects of area and environmental variables were removed, average correlations among habitat groups were low or moderate (Figure 2). It suggests that the underlying ecological and evolutionary processes might be disparate among habitat groups (Figure 2). However, the precise ecological and evolutionary processes that underpin spatial patterns in species richness are difficult to clarify based on this study's information. Therefore, further work about species traits and ecological interactions should be carried out to clarify cross‐taxon congruence (Dehling et al., 2014; Westgate et al., 2017).

**Figure 4:**
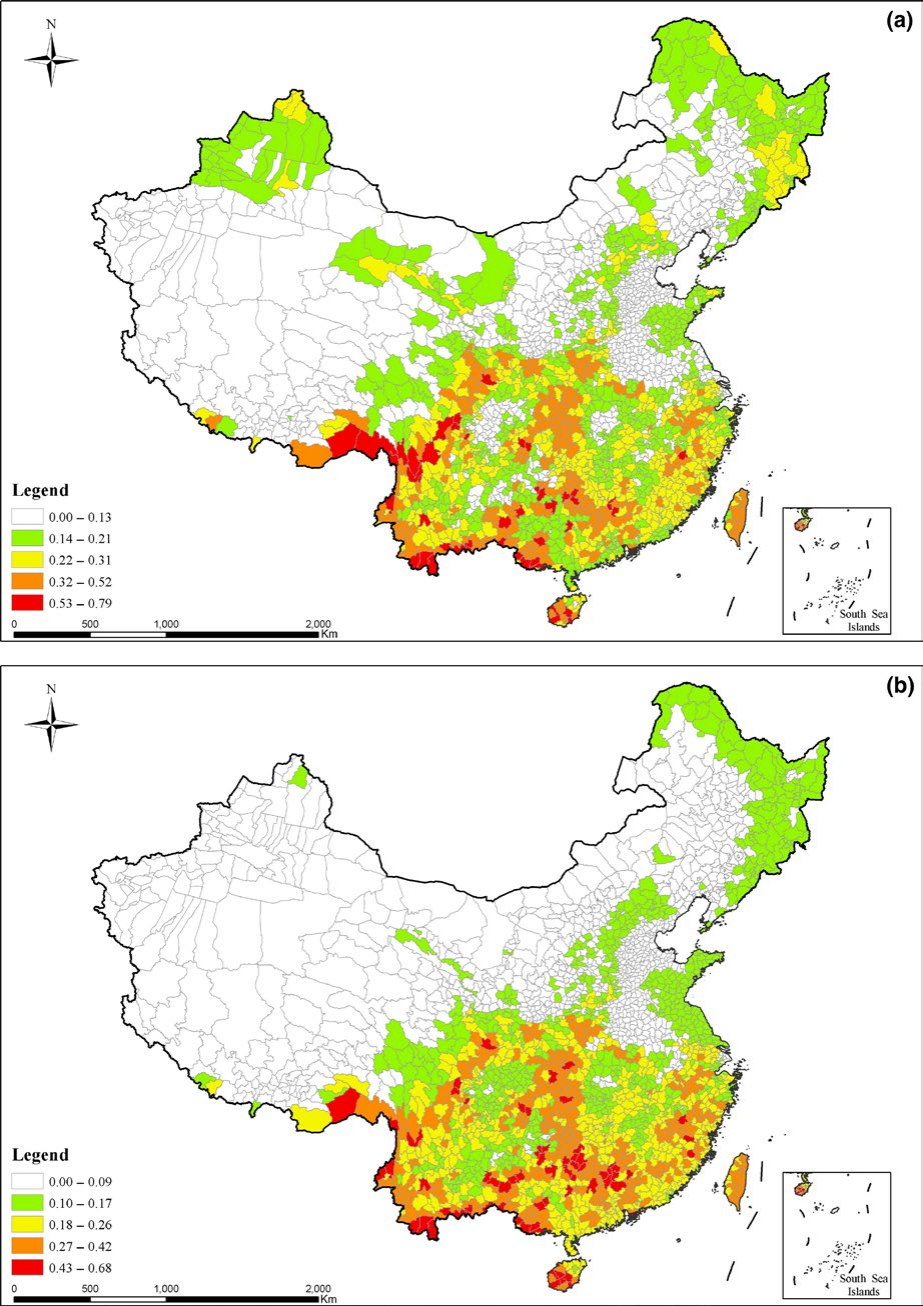
Mean species richness and hotspots across taxa and habitat groups. (a) Mean species richness across mammals, resident birds, reptiles, amphibians and vascular plants, richness data being normalized; (b) mean species richness across forest‐ and shrub‐dependent (FS) groups, richness data of habitat groups being normalized. FS groups predominantly contribute to the spatial patterns of overall species richness

Different habitat groups made different contribution to general patterns of species richness. We found that the major FS groups predominantly contribute to the spatial patterns of overall species richness (Figure 4). Based on equation (1), we produced overall species‐richness pattern by averaging species richness across vascular plants and vertebrates (Figure 4a). We also obtained species‐richness pattern for FS groups (Figure 4b) by averaging species richness across FS habitat groups based on equation (2). These two figures are very similar. In addition, some minor and rare groups were also identified, such as IUCN Red List endangered mammals *Equus kiang*,* Gazella subgutturosa,* and *Ochotona iliensis*. Such groups exhibit unique patterns and deserve special attention. However, such minor and rare groups might be missed in the overall pattern of species richness and ignored in the conservation actions. It suggests that identification of spatial patterns and conservation priorities should be based on different habitat groups from multiple taxa (Grenyer et al., 2006; Xu et al., 2015).

In summary, our results confirm previous findings that species‐richness patterns are the overlaid response of different groups to diverse environmental and evolutionary factors (Carnicer & Diza‐Delgado, 2008; Terribile, Diniz‐Filho, Rodríguez, & Rangel, 2009). Understanding of the status and trends of species‐richness patterns benefits from habitat groupings (Xu et al., 2015). Biodiversity conservation based on overall species richness alone might miss valuable biodiversity components. Our findings suggest that appropriate groupings based on habitats could reveal valuable patterns of richness gradients for conservation policy making and actions. Conservation strategies that consider multiple habitat groups from different taxa will be more effective in protecting biodiversity.

## DATA ACCESSIBILITY

The data supporting the findings of this study are available within the article and the Supporting Information.

## AUTHOR CONTRIBUTIONS

Haigen Xu, Yun Cao, and Mingchang Cao designed the study and developed the methods; Mingchang Cao, Jun Wu, Yi Wu, Zhifang Le, Peng Cui, Jiaqi Li, Fangzhou Ma, Li Liu, Feilong Hu, Mengmeng Chen, and Wenjun Tong collected the data; Yi Wu and Zhifang Le conducted the analyses; Haigen Xu and Yun Cao wrote the article.

## CONFLICT OF INTEREST

None declared.
